# A Case of Battey’s Operation with a History of the Case Prior and Subsequent to the Operation

**Published:** 1884-08

**Authors:** S. H. Gray

**Affiliations:** Barnesville, Ga.


### THE ATLANTA MEDICAL AND SURGICAL JOURNAL.

VOL. I. AUGUST, 1884. NO. 6.

### Original Communications.

### A CASE OF BATTEY'S OPERATION, WITH A HISTORY OF THE CASE PRIOR AND SUBSEQUENT TO THE OPERATION.

REPORTED BY S. H. GRAY, M. D., BARNESVILLE, GA.

Was first called to see Miss M. B——, living in a healthy country district in Monroe county, Ga., in May, 1875. Found her prostrate, exceedingly anæmic, suffering from severe facial neuralgia. The pain soon extended along the entire left side; intense pain along the lower limb, which seemed to be worse below the knee. She had no appetite; bowels were constipated. She assured me that she sometimes went as long as two weeks without having a single alvine discharge. There was dropsical effusion in the lower extremities. She had had no medical attention.

She stated that menstruation began at twelve years of age; that she had menstruated regularly and naturally until she was about fourteen years old, when, being out from home, she was overtaken by a severe rainstorm, and was thoroughly drenched and chilled. She was at this time menstruating, the flow, as usual, being natural and painless. It ceased abruptly, causing her considerable suffering.

From this time the menstrual discharge, though making its appearance regularly, began gradually to diminish in quantity, each return of the period bringing with it less and less flow, while at the same time it brought with it increased pain in the back and in the pelvic organs. Her condition continued to grow worse, until when I was called to see her, three years later, at the age of seventeen, she was suffering this intense neuralgia already referred to, besides constant pain in back and pelvic regions, all of which was very much augmented by each return of the period, when there would be only a slight stain, a very scant, dark, grumous discharge; a mere show, which lasted only two days.

I might state just here that there seems to be some scrofulous taint or diathesis in her family. She had an older sister who died at the age of twenty with tuberculosis; and from what I can learn one of her parents must also have died of tubercular disease. The appearance of her brothers and sisters now living indicates some bad constitutional taint. I mention this *en passant*, as these things may have some bearing on the subsequent history of the case. Our patient says, however, that she was never sick, but was perfectly healthy until after having been exposed to the rain-storm while menstruating.

The patient is a chaste, virtuous maiden. I hoped to be able to restore her to health by a good tonic course, relying chiefly upon iron and quinine. Under this course of treatment, kept up for a few months, color returned to her pale cheeks, the muscles were toned up in a great degree, and for some time she was comparatively free from the neuralgia; the bowels performed their functions regularly, and she was very much more comfortable in every way. She continued, however, to suffer very greatly with each return of the period.

This state of things continued during the first year of treatment. I had, however, varied the treatment from time to time, giving her the various remedies then recommended for such cases. In May of 1876, the patient having been under treatment for a year, I suggested an examination of the uterus. Her consent was obtained. On introducing the finger, I found the os uteri pointing in the direction of the symphisis pupis, the fundus lying far back in the *cul de sac*, and flexed on the cervix. I found the parts brought into view by the speculum, in a high state of congestion, with some hyperesthesia, especially in the ovarian regions; there was also copious leucorrhœal discharge from the vagina. Pressure upon the ovaries, made either through the vaginal or abdominal walls, gave her great pain.

After having been under treatment for three years, and a sufferer for five long, weary years, the patient is now no better; on the contrary, except during the first few months of treatment, she has grown worse and worse, until her condition is now almost intolerable.

She said to me to-day (Friday) that last Monday, the day for a return of the period, she suffered more than in all her previous life. To-day she is up.

Up to this date (May, 1878), I have given her both general and local treatment. The general treatment you have; the local treatment, begun two years ago, consisted of Dr. Thomas's plan of applying iodine to the endometrium, painting os and cervix uteri with tinct. iodine, using glycerine on cotton, the warm vaginal douche, with astringent douches, etc. In using sound, I find depth of uterus to be one and three-quarter inches. The internal uterus, from os to fundus, highly sensitive. After having reduced the hyperæsthesia of mucous surface somewhat, I used various pessaries in order to keep the uterus in proper position, hoping that I might thereby add somewhat to the comfort of my patient. In a very great measure I succeeded in this. So long as a well-fitting instrument was worn the patient could be on her feet without having to suffer from the discomfort of a retroverted and an engorged womb; but as soon as the instrument was removed the fundus uteri took its accustomed place posteriorly. The instrument which seemed to answer best was Hurd's retroversion pessary.

You remember the patient has now been a sufferer for about six years; under treatment for three years; her present condition, one of great suffering; suffers at different times with neuralgia of every organ in the whole system. All these sufferings are very greatly augmented by a return of the periodical evolution; tenderness over entire pelvic region, worse directly over the ovaries.

At this stage of the case, I wrote Dr. Robert Battey, of Rome, Ga., giving him a history of the case, and asking his opinion as to treatment. The doctor very kindly wrote me, stating that, "in my own experience, and in the far greater experience of Dr. Sims, such cases of long standing, chronic ovaritis are incurable, except by my method. I think it reasonably certain that she could be cured by my operation."

I read the letter to the patient; told her of the dangers attending such operations. She asked that I write again, asking the doctor the death-rate. This he very kindly furnished. Having all the lights before her, she chose the operation.

In the afternoon of November 17th, 1878, in the presence of Drs. G. M. McDowell and H. Perdue, of Barnesville, Dr. B. F. Rudisill, of Forsyth, Dr. T. F. Weaver, of Collinsville, Ala., and the writer, the patient was etherized, and Dr. Battey performed the operation. by the vaginal section. The ovaries were readily brought down through the opening, each one being secured by a strong ligature as it was brought down. The ecrasueur was used to sever the organs from their connection. Both ovaries were entirely removed; not a particle was left behind. Not an ounce of blood was lost during the entire operation.

The right ovary was of a dark purple hue—had evidently undergone decided anatomical changes. The left, though somewhat like the right, was not so unhealthy in appearance. Patient vomited several times during operation. Having had great trouble with the stomach, this organ proved to be unusually susceptible to the unpleasant or nauseous effects of the ether. There was very little shock. Patient reacted well. Operation was concluded at 4 P. M. As soon as the patient was put to bed, twenty-five drops of McMunn's elixir of opium was given per rectum. Patient continued to vomit. At 9 P. M. temperature normal, pulse 85. As she was not disposed to sleep the opium was repeated per rectum.

Nov. 18—8 A. M.—Temperature, 100; pulse, 90. Patient complains of severe pain in the pelvis, hips and loins, of same character as pains attending periodical evolutions. It is necessary to state that she had usually been forewarned of the approach of periods by the increased severity of the pains. This had been the case for two or three days previous to the operation. Nausea continues, for which the patient was given lime water and milk, with ice, ice-water, iced whiskey, etc.

3 P. M.—Temperature, 102½; pulse, 112. Still suffers pains of same character. Nausea and vomiting continue. Relieved bladder with catheter. Give elixir opium, seventy-five drops, per rectum.

10 P. M.—Temperature, 103½; pulse, 115. Nausea continues with occasional efforts at vomiting, causing great pelvic pain.

Nov. 19—3 A. M.—Temperature, 104; pulse, 115. Patient more quiet; very little nausea. 6 A. M.—Patient has been more comfortable since last report than at any time since operation. Pulse and temperature about the same.

4 P. M.—Temperature, 105; pulse, 120. Nurse reports patient as resting well up to 12 M. Since that time, however, she has had more nausea with efforts at vomiting, though nothing has been ejected from the stomach.

5 P. M.—Temperature, 105 ; pulse, 140. I ordered sulph. quinia, gr. x—repeated in two hours. Nausea coming on so distressingly, no more quinine was given until 10 P. M , when gr. xx, per rectum was given. 12 o'clock at night, temperature, 102½ ; pulse, 120.

Nov. 20—4 A. M.—Temperature, 101; pulse, 118 Has had good deal nausea, but slept some. Asks for mixture which she had been taking for several weeks previous to operation – equal parts paregoric and tinct. valerian. After giving her two teaspoonsful of this, nausea was less, and she was decidedly more comfortable.

6 A. M.—Temperature, 100; pulse, 100. Does not complain of much soreness. Quinine, gr. x given per rectum.

5 P. M.—Nausea has been excessive during day with but little vomiting. Eructations of gas with distressing heaving and retching, causing great pressure on pelvic organs ; good deal of soreness and pain in ovarian regions—more on right side than on the left. Has taken milk and chicken broth occasionally through the day.

10 P. M. – Pulse, 105 ; temperature, 103. Gave quinine, gr. x ; elixir opium, seventy-five drops, per rectum. Great pain in region of ovaries. Excessive tenderness under pressure made over hypogastric and iliac regions.

Nov. 21—5 A. M.—Pulse, 93 ; temperature, 102. Patient had a better night than any since operation. Has taken little warm milk and tea occasionally ; also, a sip of water when called for. Had copious perspiration during night. Gave, per rectum, quinine, gr. x ; dovers powder, gr. x. Directed quinine, gr. iv, every four hours, to be given per rectum.

5 P. M.—Temperature, 102¼ ; pulse, 95. Patient slept a good deal. Takes milk and broth in very small quantities. No pain except from being moved or from vomiting, which has occurred twice in twenty-four hours. Great pelvic tenderness, still on right side. Gave, per rectum, quinine and dover powders, equal parts gr. x, and directed quinine to be continued as before.

Nov. 22 – 10 A. M.—Temperature, 102 ; pulse, 86. Patient slept well during night. Complains of severe pain in region, extending into shoulder. Complained of this very greatly at times for last year or two. Directed calomel, gr. ⅙, bis. sub nit., gr. ij to be given every hour—quinine, gr. iij, per rectum, every four hours. For two days had not complained of *old* pains in ovarian regions.

Still, very great pelvic tenderness, though somewhat less than at last report. Ordered large sinapisms of mustard over liver.

Nov. 23—8 A. M.—Patient had a good night. But little nausea since beginning with calomel and bismuth. Had copious alvine discharge at 10 P. M. Another good evacuation at 6 A. M. to-day. After which gave elixir op.um, gr. xxv, per rectum. In reply to my question, "How are you getting on this morning?" promptly replies, "I am better." Has had but little pain over liver and shoulder. Relishes milk.

Nov. 24—8 A. M.—Patient passed tolerably comfortable day until late in the afternoon, when the bowels began to act, and notwithstanding the fact that the nurse followed each action with injection of opium, there were six discharges within a few hours' time. Patient slept well. Calls for food. Not so much soreness in pelvic region generally, but very great pain in region of right ovary. Pain rather paroxysmal, extends from right iliac region along the entire side to the shoulder. She says of same character as her *old* pains during her menstrual period. Left side giving no trouble.

Nov. 25—8 A. M.—Temperature, $100\frac{1}{2}$; pulse, 85. Patient has done well since last report. Still, pain along right side, though not so severe. Had opium once in twenty-four hours. Is bright and cheerful. Appetite good. Digestion good. But little pelvic soreness. Continued quinine in less quantity.

Nov. 26—11 A. M.—Temperature, $100\frac{3}{8}$; pulse, 84. Patient suffered but little since last report. Upon pressure in right ovarian region found some tenderness with possibly slight tumefaction. Entire left side free from pain for the first time in years.

Nov. 27—11 A. M.—Patient had good night. Is bright and cheerful. Had no opiate for forty-eight hours. Bowels move naturally. Temperature, $100\frac{1}{4}$; pulse, 83.

Nov. 28—2 P. M.—Patient complains of being very tired. Appetite continues good. Temperature, $100\frac{1}{2}$; pulse, 83.

Nov. 30—8 A. M.—Temperature, $100\frac{3}{8}$; pulse, 93. Less soreness in right ovarian region. Patient sits up in bed.

DEC. 2—4 P. M.—Sixteenth day after operation. Patient says the week ending yesterday was to her the most comfortable week she has spent in the last six years. To-day, however, she is suffering greatly from dysuria, with temperature, $101\frac{3}{4}$; pulse, 104. Frequent desire to micturate, with great pain just after the act. I find by vaginal touch very great tenderness of bladder and urethra with extreme sensitiveness of meatus. Direct that she have spts. nitre with fld. ext. belladonna every three or four hours.

DEC. 4—8 A. M. - Patient has but little pain in ovarian region. Still suffers greatly from micturition. Pulse, 93; temperature, 100½. Appetite good.

DEC. 6—4 P. M. —Micturition not quite so painful as at last report, though pressure upon the bladder still gives great pain whether made through the abdominal or vaginal walls. Find great sensitiveness extending from linea alba in supra-pubic region across to a point very near the right iliac fossa; at this point there is no tenderness under pressure, but just here the patient says is the seat of pain, instead of directly over the ovary as heretofore. This pain i paroxysmal and is described by her as of the same character as *the old pain.* There is occasional slight pain just over the ovary, but no throbbing pain. Temperature, 103½; pulse, 100. For two days had been taking quinine, gr. x, daily, also acetate potass and fld. ext. belladonna. Not quite so much pain following micturition. Appetite not so good. Bowels act naturally.

DEC. 7—5 P. M —Temperature, 102, pulse, 100. To-day patient has suffered good deal from pain over liver and lumbar region. Stomch not bearing nitre well, this was left off. Some less pain from nicturition, but vomiting caused severe pain in right ovarian region.

DEC. 8—4 P. M.—-Patient reports much less pain from micturition, but more in right ovarian region. There is decided fullness in this side. Patient more comfortable with thighs plexed. Some throbbing pain, not constant. Pulse, 102, temperature, 104. Patient says appetite good. Diet, bread, milk and eggs.

DEC. 9—3 P. M —Condition of right side unchanged. Temperature, 104, pulse, 100.

DEC. 10—4 P. M.—Patient reports more comfortable to-day than yesterday. Pulse, 90, temperature, 103⅗. Condition of bladder still improving; continued previous treatment with addition of warm poultices applied all over the abdomen. Also painted entire abdominal surface with tinct. iodine.

DEC. 11—3 P. M.—Less general abdominal tenderness. No apparent change in region of right ovary. Temperature, 101. Pulse, 85.

DEC. 12—10 A. M.—Temperature, 99⅘. Pulse, 82. Abdominal tenderness subsiding, also improvement in condition of parts around the right ovary; very slight pain from passing water.

DEC. 13.—9 A. M.—Temperature, 99⅞. Pulse, 82.

DEC. 17.—10 A. M.—30th day after operation. Find patient suffered a good deal last night, became nauseated; bowels have just acted, giving her considerable pain on account of hardened fæces. Great tenderness over entire pelvic region. Some general abdominal tenderness with distension, tympanitic. Temperature, 103. Pulse, 106. Parts exceedingly sensitive to rectal or vaginal touch. Give elixir opium gtts. lx. per rectum. Same treatment, both general and local, continued.

DEC. 18.—6 A. M. Patient had tolerably good night. Temperature, 101½. Pulse, 90. Condition otherwise about the same. 5 P. M.—Temperature 100. Pulse, 90. About seventy-five drops elixir opium given in twenty-four hours per rectum. Other treatment same.

Dec. 19—4 P. M.—Temperature, 99¼; pulse 75. Appetite good. Patient says just over right ovary feels as if it ought to be "opened."

Dec. 21—4 P. M.—Temperature, 99¾; pulse, 72. No change of treatment.

Dec. 22—4 P. M.—Patient has had a better day. Pulse, 70. Failed to take temperature.

Dec. 25—11 A. M.—Patient doing well. Pulse, 68; temperature, 99¼. Abdomen soft and natural to the touch. Very little tenderness except in the region of right ovary, and here the tenderness is very much lessened; very little fullness here also.

Dec. 28—9 A. M.—Patient suffering more pain. Pulse, 78.

Dec. 30—4 P. M.—Find patient nervous, tossing about the bed. Has had no opium for three days, family failing to get it as directed. In consequence of so much tossing there is more general abdominal soreness as well as more local pain. I order opium immediately.

Jan. 2—1879, 4 P. M.—Patient more cheerful. Pulse, 78. Still some fullness in right ovarian region.

Jan. 4—11 A. M.—Stomach giving more trouble; character of trouble very much the same as frequently suffered before the operation. Pulse, 75. Condition of the right side about the same. For condition of stomach order calomel, gr. ⅙; to be taken every three hours. No other medicine except opium and belladonna.

Jan. 5—5 P. M.—Patient reports stomach much better. Bowels have not acted for several days; order seidlitz to be repeated in two hours if necessary.

Jan. 7—4 P.M.—Dr. Battey, sees the patient with me, and after careful examination of the case thinks pelvic organs in good condition; finds (as I had noticed sometime before) uterus already beginning to atrophy. He is not fully satisfied as to trouble in right groin but is inclined to think it must be about the cœcum. Temperature, $99\frac{3}{4}$; pulse, natural. Dr. Battey suggests the leaving off of all medicines except opium and directs this given per rectum. Diet, milk, eggs, rice and bread with the lighter meats.

A month or two after this, when the patient began to walk about the house and yard, she found herself entirely free from that weight and dragging down pain in pelvis which had given her so much trouble for years. She had no return of the menses which had caused her such intensely agonizing pain and which had rendered life a torture. It is true that she did not at once get rid of all that train of nervous phenomena which had been preying upon her for so long a time. For several months after she was going about, walking wherever she wished, she would sometimes suffer neuralgia of some one or more of the important organs of the body.

I will mention that her eyes suffered in common with other organs. Vision was very seriously impaired on account of the great nervous disturbance produced by long continued disease of organs that play an important part in the female economy as the ovaries. The optic nerve seemed, in common with the whole system, to be weakened. She could read but a few minutes at a time and could not distinguish objects a little way off. But the eyes in common with the whole system have been improving until to-day. Nov. 28, 1880, I called to inform her that I was writing up a report of her case, and wanted to know precisely of the state of her health.

As she met me I involuntarily remarked, "really you must have forgotten that you were ever sick, you are looking so red, so rosy and so cheerful." Her reply was: "No, I haven't forgotten that I was sick, but I don't feel now as if I had ever been sick."

The patient has none of her *old pains* whatever; has had no appearance of a return of the menstrual period since the operation nearly six years ago, and is now well.

